# The Influence of Climatic Seasonality on the Diversity of Different Tropical Pollinator Groups

**DOI:** 10.1371/journal.pone.0027115

**Published:** 2011-11-02

**Authors:** Stefan Abrahamczyk, Jürgen Kluge, Yuvinka Gareca, Steffen Reichle, Michael Kessler

**Affiliations:** 1 Institute for Systematic Botany, University of Zurich, Zurich, Switzerland; 2 Faculty of Geography, University of Marburg, Marburg, Germany; 3 Coleccíon Entomologica, Museo de Historia Natural Alcide d'Orbigny, Cochabamba, Bolivia; 4 The Nature Conservancy, Bolivia Office, Torre B Multicentro, La Paz, Bolivia; Centre National de la Recherche Scientifique, France

## Abstract

Tropical South America is rich in different groups of pollinators, but the biotic and abiotic factors determining the geographical distribution of their species richness are poorly understood. We analyzed the species richness of three groups of pollinators (bees and wasps, butterflies, hummingbirds) in six tropical forests in the Bolivian lowlands along a gradient of climatic seasonality and precipitation ranging from 410 mm to 6250 mm. At each site, we sampled the three pollinator groups and their food plants twice for 16 days in both the dry and rainy seasons. The richness of the pollinator groups was related to climatic factors by linear regressions. Differences in species numbers between pollinator groups were analyzed by Wilcoxon tests for matched pairs and the proportion in species numbers between pollinator groups by correlation analyses. Species richness of hummingbirds was most closely correlated to the continuous availability of food, that of bees and wasps to the number of food plant species and flowers, and that of butterflies to air temperature. Only the species number of butterflies differed significantly between seasons. We were not able to find shifts in the proportion of species numbers of the different groups of pollinators along the study gradient. Thus, we conclude that the diversity of pollinator guilds is determined by group-specific factors and that the constant proportions in species numbers of the different pollinator groups constitute a general pattern.

## Introduction

Animal pollination is one of the key ecosystem services in natural habitats as well as in many agricultural systems [1], [2]. Currently, these services are threatened by habitat destruction and climate change, as illustrated by marked decreases in pollinator diversity and abundance in different parts of the world [3], [4], and further declines in species numbers of bees, bumblebees, and butterflies are predicted as results of future landuse and climate change [5], [6], [7], [8]. These declines will have severe consequences for the ecosystem services provided by pollinators. In Western Europe, for example, obligate animal-pollinated plant species have declined in parallel with their pollinators [1]. Among animal pollinated plant species, particularly those with the most specialized pollination requirements, are expected to become at least locally extinct [9]. However, also generalists that are well integrated into asymmetric and nested interaction networks that are in general buffered against disturbances, can be negatively affected if the networks reach a tipping-point and collapse [10], [11].

In tropical forests, up to 99% of all plants are dependent on animal pollination [12]. Tropical areas host a large variety of diurnal and nocturnal pollinator groups. The main diurnal pollinator groups in South America are bees and wasps, flies, beetles, butterflies, and hummingbirds. At night, they are mainly replaced by moths and bats. Overall, bees are the most important pollinators worldwide [13]. In two Amazonian rainforests 54% of all plant species are pollinated by bees [14]. Vertebrates, mainly birds and bats, pollinate 3–15% of the plant species in the tropics and subtropics [15], [16].

The crucial factors determining diversity, composition, and temporal variability of the pollinator assemblages are climatic seasonality, and spatial and temporal variation of food resources [17], [18]. Most vertebrate pollinators need a continuous supply of food due to their high metabolic rate, long lifespan and homoeothermic bodies. In turn, these enable them to visit flowers even during cool and rainy weather when insects are unable to fly. Therefore, large-scale diversity patterns hummingbirds are in line with the general pattern of high tropical diversity [19], peaking in warm and humid regions. Previous studies suggest that hummingbirds are more diverse in humid rainforests than in deciduous forests [20], [21] because of a less pronounced climatic seasonality. Butterflies show a similar diversity pattern as hummingbirds. However, for butterflies the absence of low temperatures is most important [17]. Instead, bees are more diverse in warm, temperate, and xeric regions than in the humid tropics [22], [23], presumably reflecting their physiological and behavioural adaptations. In contrast to hummingbirds, insects such as bees and wasps or butterflies are able to survive long phases of unfavourable conditions in larval stages or hibernating as adults. Vertebrate pollinators such as hummingbirds or nectar-feeding bats and even some butterfly species can avoid areas with unfavourable conditions during parts of the year by conducting local and regional migrations following shifting food resources [24], [25], [26], [27].

Comparing the diversity patterns of the different pollinator groups, the proportion of hummingbird species should be higher in per-humid forests whereas the proportion of insect pollinators should be higher in deciduous dry forests with a marked climatic seasonality. However, cross-taxon comparisons are difficult because the majority of studies on large-scale patterns of pollinator diversity to date have only focussed on single pollinator groups and have not considered the relation of pollinators to their food plant species [17], [20] or have been conducted on oceanic islands, where pollinator diversity is reduced [18]. Independent of the study area, studies on a smaller scale considering the relation to food plant species of single groups of pollinator have shown that hummingbird diversity is mainly influenced by a continuous availability of food [28], [29] whereas the number of bee and wasp species is determined by the number of flowers and the diversity of food plant species [30].

Little is known about seasonal changes of different pollinator guilds at a given locality. In particular, studies along climatic gradients are completely lacking. Studies considering single groups of pollinators suggest that the seasonal variation in species richness of butterflies as well as of bees and wasps [31], [32], [33], [34] is more pronounced than in hummingbirds [29] because insect pollinators can outlive phases of unfavourable environmental conditions in larval stages or hibernating as adults. For example, in Venezuela, a higher species numbers of insect pollinators during the dry season is known when rain does not restrict their activities too much [35]. The lack of comparative studies considering several pollinator groups severely limits our understanding of the impact of climatic conditions, particularly climatic seasonality.

This lack of knowledge is particularly important when predicting effects of habitat and climate change on pollinator diversity. For large parts of the tropics, including central South America an increase in temperature and a decrease in precipitation is predicted [36], [37]. Our study was directed at addressing this knowledge gap by exploring the relations of the diversity between the three main diurnal pollinator groups (bees and wasps, butterflies, hummingbirds) to climatic factors as well as to flower number and food plant diversity along a natural gradient of precipitation and climatic seasonality in tropical and subtropical Bolivian forests. This region, located at the southern margin of the Amazon basin presents a good opportunity to assess how pollinator assemblages change along a climatic gradient mimicking the changes predicted for the next decades [37]. The study was designed to test the following three hypotheses:

- Species richness and abundance of pollinator groups is more strongly determined by food-related than directly by climate-related factors (although climate certainly influences food availability).
- Species richness and abundance of insect pollinators shift between seasons whereas that of hummingbirds does not show marked seasonal changes.
- Towards areas with lower climatic seasonality, pollinator assemblages include a higher proportion of hummingbirds and their food plants relative to insect pollinators and their food plants.

## Materials and Methods

Our study complies with the current laws of Bolivia and Switzerland and with international rules. Permissions for fieldwork in Bolivia and collecting and exporting samples have been provided by national and local authorities.

### Study sites

**Figure 1:**
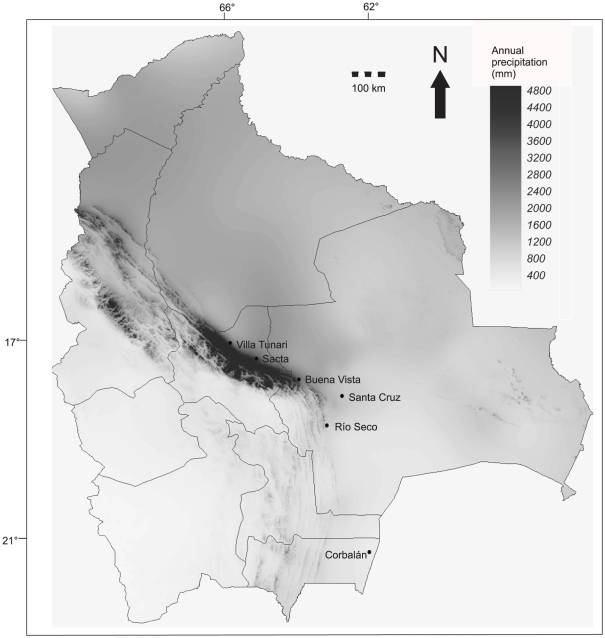
Precipitation map of Bolivia (based on [**38**]) showing the six study sites (circles) and the provinces.

Our study was conducted at six sites in central and southern Bolivia: three non-seasonal, tropical (Villa Tunari: 16°57′59 S, 65°24′44 W; Sacta: 17°06′03 S, 64°47′02 W; Buena Vista: 17°30′49 S, 63°38′16 W) and three seasonal, subtropical sites (Santa Cruz: 17°46′48 S, 63°04′02 W; Río Seco: 18°42′44 S, 63°11′35 W; Corbalán: 21°36′15 S, 62°27′45 W; Figure 1). Along this latitudinal gradient, mean annual precipitation decreases from 6258 mm at Villa Tunari to 410 mm at Corbalán, while seasonality in temperature and precipitation increase [38] (Table 1). All study sites consisted of primary, occasionally slightly disturbed forest and lay between 200 m and 440 m elevation. All sites are part of a larger forest system extending from Amazonia to the Gran Chaco.

**Table 1:** Environmental characteristics of the study sites.

	Elevation (m)	Annual precipitation (mm)	No. arid months	Temperature mean (°C)	Minimum temperature (°C)	Temperature amplitude (°C)
Corbalán	268	410	8	25.2	−3	7
Río Seco	434	729	6	25.0	−1	5
Santa Cruz	397	1166	2	25.2	1	5
Buena Vista	424	2000	0	25.3	3	4
Sacta	204	3457	0	26.7	5	4
Villa Tunari	400	6258	0	26.6	6	3

### Environmental data

We extracted the climatic data from SAGA, a climate model for Bolivia based on an empirical modelling scheme [38]. At each site we took four soil samples from the non-organic soil horizon. The samples were air-dried and later analyzed for pH, C/N-proportion, cationic exchange capacity, and base saturation.

### Field sampling

Each locality was visited twice for 16 days between November 2007 and October 2008, once during the rainy season (November to April) and once during the dry season (May to October). Such a short survey time is liable to result in incomplete sampling, especially of insects [39], [40]. On the other hand, since sampling at the different sites could not be conducted simultaneously, sampling was spread over three months, so that sites were visited at different times during each season. This too might influence our perception of the plant and pollinator assemblages. Because these two limitations are counteracting (longer sampling per site increases temporal differences between sites), we decided on the 16-day sampling routine as a compromise between both potential problems. Further, we did not include replicate study sites in each study region, again as the result of a compromise between sampling completeness and number of study sites.

At each site, we established a 1.5 km long study transect along a path through the forest with a minimum distance to the forest border of 150 m to avoid edge effects [41], [42]. Each transect was visited for 13 continuous days between 7∶30 am and 15∶30 pm. On the remaining three days, we visited a 350 m long section of the forest border because some pollinator species normally live in the tree crowns and only come down to lower vegetation levels along forest edges.

All animal-pollinated plant species flowering three meters to both sides of the transect were recorded [43] and for all species the number of flower was estimated using five categories: 1–10, 11–50, 51–200, 201–1000 and 1000+ flowers. For further analyses, we defined the “number of plant species” as the number of all plant species used by a certain pollinator group that were flowering along a transect. The number of plant species was counted for each season (dry and wet) separately and added to the total number of plant species. The “number of flowers” was summed from the minimum values per category of plant species used by a certain pollinator group that were flowering along a transect. These values were only expressed per season.

We recorded all plant species visited by hummingbirds (pers. obs.) or showing the anatomical adaptations to hummingbird pollination [44] as food plant species of hummingbirds. According to our field observations, all animal-pollinated plant species providing nectar or pollen during the day were recorded as food plants for bees and wasps, irrespectively of their morphology. Plant species showing the typical anatomical adaptations to hummingbird pollination were thus also counted as “bee and wasp plants” because bees were regularly observed stealing nectar or pollen from them (pers. obs.). All plant species providing nectar during the day, regardless of visits by other pollinator groups were categorized as “food plant species of adult butterflies”, because butterflies are also known to be opportunistic in their food choice [45].

We divided the diurnal pollinators with the exception of flies, which were not considered in our study, into three main pollinator groups [following 46]: bees and wasps, butterflies, and hummingbirds. We treated all bees and wasps as potential pollinators because several studies show that even species that do not feed their offspring with pollen use nectar as an energy source during adult stage [47], [48]. We distinguished flower-visiting and hence pollinating butterfly species from species not visiting flowers (i.e., species feeding on fruits, dung, or not at all as imagines) based on field observation, literature, and expert knowledge.

For pollinator sampling, we first recorded all diurnal pollinators visiting the plants along our study transects during four days, watching each flowering plant species several times for 15 min at different times of the day. Frequency and length of pollinator observations on different plant species depended on the abundance of the respective species along the transects. Bees and wasps and butterflies visiting flowers were captured with nets. Further bees and wasps were collected during five days using 13 pairs of yellow and blue pan traps situated next to each other along the transects [49]. Euglossine bees were collected for two days using 39 modified McPhail traps with 13 different odour baits [50]. With both trap types it was possible to estimate the numbers of individuals per species. Afterwards, for three days we captured flower-visiting butterflies with nets, focusing on recording as many species as possible but not collecting every individual seen (which would have been impossible). Further, for three days hummingbirds were recorded at their food plants. Additional hummingbird observations were made while walking on the transects. No direct individual counts were attempted of hummingbirds due to their high mobility, but we estimated the abundance of the recorded hummingbird species in three categories: 1–3 (rare species), 4–9 (occasional species) and 9+ individuals seen per survey (common species) [29]. Thus, we are able to use estimates of individual numbers for bees and wasps and hummingbirds in our analysis but not for butterflies.

We were unable to conduct formal estimates of sampling completeness [51] because this requires counts of numbers of individuals per species which were not available for most of our taxa. However, for those groups for which regional species counts are available, our sampling is reasonably complete: hummingbirds: 25 species known to occur in the study area [52], 21 (84%) species detected by us; euglossine bees: 68 species known for Amazonia [35], 48 (71%) collected in our study; butterflies: 96 nectar-feeding species known for the Botanical Garden Santa Cruz [53], 69 (72%) collected in our study. Furthermore, published local studies show similar patterns to those detected by us [25], [54], [55]. Thus, considering that our sampling effort lies within the range of studies sampling more than one site (8–92 days, as estimated in [56]), we consider that our sampling is complete enough to reveal general patterns in each group of pollinators.

Hummingbirds, butterflies, euglossine bees, and plants were identified to species level using the relevant literature [57], [58], [59], [60] and with the help of experts (butterflies: O. Mielke, K. Willmott, R.K. Robbins; euglossine bees: P. Gottleuber). The remaining bees and wasps which made up about 90% of the total group species richness were identified to family level using [61] and then arranged into morpho-species. The use of morpho-species is widely accepted to assess insect species richness in the tropics because of the large number of total and undescribed species of insects in this area, the large number of collected individuals (4328 in our case), and the widely missing identification literature [62].

### Data analysis

All analyses were conducted separately for each of the three pollinator groups. Our estimates of the numbers of flowers of food plants as well as of the numbers of hummingbirds were only rough estimates; for butterflies we did not obtain abundance data at all. To circumvent these limitations, we conducted all relevant analyses both with abundance and presence-absence data.

To test for differences between groups we used Wilcoxon rank tests and Wilcoxon tests for matched pairs due to non-normality in our dataset.

To assess latitudinal trends in species richness of the three pollinator groups, linear regressions of species numbers of the different pollinator groups against the latitude of the study sites. Due to conspicuously lower values in species number for all pollinator groups at the northernmost site Villa Tunari, in a second round of analyses we excluded this site. Further, we compared species numbers between the three northern (non- to slightly climatically seasonal) and southern (strongly climatically seasonal) sites by Wilcoxon rank tests.

To test hypothesis I, we conducted linear regressions to assess the influence of individual explanatory variables (plant species number, flower number, climate and soil related parameters) on individual pollinator groups (individual and species numbers). Because we only had six study sites, we refrained from calculating multiple regression models. To relate seasonal differences in species and individual number to environmental factors of climate and food seasonality, we conducted linear regressions between the difference in species respectively individual number per site between seasons and factors of seasonality.

To test hypothesis II, we tested differences of species numbers of the three pollinator groups between seasons and differences in bee and wasp and hummingbird abundance between seasons with Wilcoxon tests for matched pairs. We used Wilcoxon tests for matched pairs to find out whether the proportions of species per pollinator group occurring only at a single site during one season differed between seasons.

To test hypothesis III, we used correlation analyses to compare the abundances between the three pollinator groups, between flower numbers, and between numbers of plant species utilized by the three pollinator groups along the seasonality gradient.

Unless indicated otherwise, all calculations were performed with the statistical platform R [63]. Following Roback and Askins [64] we did not use Bonferroni corrections to correct for the high number of tests. Further, we accepted an additional level of marginal significance at p≤0.1 because of the low number of study sites. The marginally significant results did not change our overall perception of the patterns, but it helped us to take in account additional, potentially important factors that would have been ignored by only looking at the significance level of p≤0.05.

## Results

Overall, we recorded 21 species of hummingbirds, 513 morphospecies of bees and wasps, 243 species of diurnal, nectar-feeding butterflies, and 168 species of flowering food plants (Table 2). Due to regular pollen and nectar thievery, all plant species were used by bees and wasps. Further, 143 plant species were used by butterflies and 45 by hummingbirds (Appendix 1).

**Table 2:** Species richness of pollinator groups at each study site during the rainy (R) and the dry season (D) and in total.

	No. of	Bees and wasps			Butterflies			Hummingbirds		
		R	D	total	R	D	total	R	D	total
Corbalán	Pollinator sp.	88	64	119	25	28	40	3	3	3
	Flowers	1022	1597	-	1022	1597	-	368	1076	-
	Plant sp.	22	18	35	22	18	35	8	6	11
Río Seco	Pollinator sp.	90	94	149	28	38	50	3	3	5
	Flowers	684	1096	-	420	1095	-	201	315	-
	Plant sp.	14	16	29	10	15	24	1	5	5
Santa Cruz	Pollinator sp.	62	78	116	36	50	69	3	2	5
	Flowers	891	768	-	602	633	-	62	104	-
	Plant sp.	21	18	33	12	13	23	2	4	5
Buena Vista	Pollinator sp.	122	142	208	31	69	88	9	6	11
	Flowers	2858	1653	-	1379	1590	-	1053	1467	-
	Plant sp.	20	14	28	10	11	18	3	7	8
Sacta	Pollinator sp.	115	99	173	37	61	77	8	6	11
	Flowers	2096	575	-	1883	523	-	1138	420	-
	Plant sp.	26	-15	32	24	13	28	8	10	12
Villa Tunari	Pollinator sp.	73	75	120	38	50	74	5	4	6
	Flowers	1043	800	-	991	778	-	149	268	-
	Plant sp.	23	19	31	21	18	29	8	8	11

A comparison of species numbers between the three less seasonal northern and the three more seasonal southern sites revealed marginally significantly higher numbers in the north for hummingbirds (Wilcoxon rank test: W = 9, p = 0.072) and butterflies (W = 9, p = 0.10) but not for bees and wasps (W = 8, p = 0.20).

The total number of butterfly species (linear regression, R = −0.87, p = 0.057) decreased latitudinally from non-seasonal Sacta to seasonal Corbalán, while the total number of bee and wasp species (R = −0.58, p = 0.306) and hummingbird species (R = −0.77, p = 0.126) did not show a significant latitudinal pattern. Separated by seasons, we observed marginally significant latitudinal decreases for the species numbers of butterflies during the rainy (R = −0.84, p = 0.073) and during the dry season (R = −0.87, p = 0.055). Hummingbirds (rainy season: R = −0.61, p = 0.276; dry season: R = −0.49, p = 0.402) and bees and wasps (rainy season: R = −0.31, p = 0.612, dry season: R = −0.64, p = 0.246) did not show a significant pattern during single seasons. For hummingbirds as well as bees and wasps, the northernmost, wettest site at Villa Tunari had a conspicuously lowered species richness compared to the nearest locality (Sacta). The species numbers of food plants as well as of flowers utilized by the different pollinator groups did not show any significant trends along the latitudinal transect (Table S1).

Linear regression analyses between the species numbers of each pollinator group and biotic and abiotic explanatory factors revealed that the species number of hummingbirds was most strongly related to the number of flowers during the rainy season (Table 3). The number of butterfly species showed negative relations to the annual temperature amplitude and to the number of arid months as well as positive relations to the annual minimum temperature. The total number of bee and wasp species was most strongly related to the number of flowers during the rainy season. In the dry season, the number of bee and wasp species was negatively related to the total species number of food plants as well as to the species number of food plants flowering during the dry season. In the rainy season, species number of bees and wasps was related to the total species number of food plants and to the species number of food plants flowering during the rainy season. In hummingbirds and butterflies the significant relations to environmental factors for the species numbers per season agreed with the relations for the total species numbers (Table 3). For the number of individuals of bees and wasps and hummingbirds we found similar but less strong relations as for species numbers (Table S2).

**Table 3:** Strength of relation (R-values) for pair-wise regression analyses of the species numbers of the three pollinator groups during rainy season (R), dry season (D) and in total, against all biotic and abiotic environmental factors considered; - not analysed, ^ p≤0.1, * p≤0.05, ** p≤0.01.

	Bee and wasp sp.			Butterfly sp.			Hummingbird sp.		
	R	D	total	R	D	total	R	D	total
Elevation (m)	−0.30	−0.28	0.02	−0.04	0.09	0.16	−0.20	−0.19	−0.30
Annual precipiation (mm)	−0.05	−0.03	−0.03	0.76̂	0.41	0.55	0.37	0.38	0.40
Temperature mean (°C)	0.16	−0.06	−0.06	0.74̂	0.42	0.49	0.48	0.55	0.47
Temperature amplitude (°C)	−0.15	−0.42	−0.35	−0.80̂	−0.74̂	−0.82*	−0.60	−0.58	−0.53
Minimum temperature (°C)	0.19	0.32	0.30	0.88*	0.76̂	0.84*	0.66	0.62	0.68
No. arid months	−0.64	−0.50	−0.43	−0.86*	−0.91*	−0.97**	−0.68	−0.73̂	−0.61
Flower no. R	0.12	-	0.81*	0.31	-	0.53	0.91*	-	0.92**
Flower no. D	-	−0.89*	0.62	-	−0.23	−0.29	-	0.48	0.48
Food plant sp. no. total	0.83*	0.81*	0.70	−0.20	−0.70	−0.65	0.36	0.41	0.53
Food plant sp. no. R	0.85*	-	0.03	0.23	-	0.13	0.06	-	0.23
Food plant sp. no. D	-	0.32	−0.53	-	−0.77̂	−0.66	-	0.60	0.72

Linear regression analyses between the species number of pollinator groups against soil parameters hardly recovered any significant results. They are therefore not discussed further (Table S3).

When we related seasonal differences in species and individual number to environmental factors of climate and food seasonality we found that the seasonal difference in hummingbird species number was best explained by the number of arid months while the difference in bee and wasp abundance was best explained by annual temperature amplitude (Table S3).

**Figure 2:**
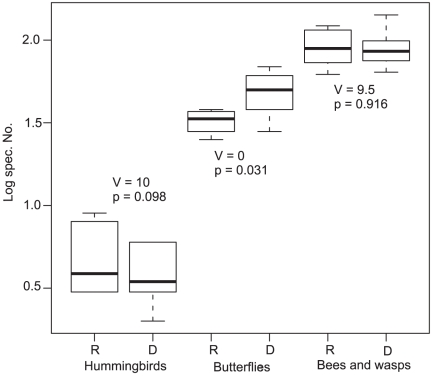
Wilcoxon tests for matched pairs between number of species per pollinator group during the rainy (R) and dry (D) season. Box plots show the median values (thick lines), second and third quartiles (box margins) and 95% confidences intervals (whiskers).

Butterflies showed a significantly higher species richness during the dry than during the wet season, while for hummingbirds we obtained only a marginally significant result and for bees and wasps we did not obtain a significant result (Figure 2). For the abundance of bees and wasps (V = 16, p = 0.313) and hummingbirds (V = 5, p = 1.00) we did not detect significant differences between seasons. We further found that the proportions of species per pollinator group occurring only in a single site during one season was significantly higher for butterflies during the dry season (V = 0, p = 0.031) and did not show significant differences for bees and wasps (V = 0, p = 0.181) and hummingbirds (V = 5, p = 0.313).

We found a positive correlation of the number of hummingbird species to those of butterflies as well as bees and wasps (Table 4). The correlations of the species number of pollinator groups separated by seasons revealed significant positive results always just for one season per pollinator group (Table 4). Correlation analyses of the total number of flowers used by the different pollinator groups detected even stronger relations compared to the species number of pollinator groups. The total numbers of flowers used by the different groups of pollinators were all positively correlated with each other. We found similar results for each season separately (Table 4). With a correlation of the total number of flowering species utilized by the different pollinator groups we only found a marginally significant, positive result between bee and wasp and butterfly species numbers. The correlations of species numbers used by the different groups in each season separately gave divergent results (Table 4).

**Table 4:** R-values of linear correlation analyses for the species numbers of the pollinator groups, number of flowers and flowering species used by the different pollinator groups during the rainy (R) and dry (D) season and in total; ^ p≤0.1, * p≤0.05, ** p≤0.01, *** p≤0.001.

	Bees and wasps - butterflies	Bees and wasps - hummingbirds	Butterflies – hummingbirds
Pollinator group sp. no. total	0.61	0.87*	0.84*
Pollinator group sp. no. R	−0.18	0.82*	0.34
Pollinator group sp. no. D	0.79̂	0.73	0.76̂
Flower no. used by pollinator groups R	0.78̂	0.92**	0.88*
Flower no. used by pollinator groups D	0.99***	0.89*	0.87*
Plant sp. no. used by pollinator groups total	0.79̂	0.36	0.64
Plant sp. no. used by pollinator groups R	0.80̂	0.81̂	0.97*
Plant sp. no. used by pollinator groups D	0.79̂	−0.31	−0.02

## Discussion

Overall, we found that the species number of butterflies and hummingbirds increased from south to north towards the equator, which corresponds to the well-known latitudinal gradient of species richness in these groups [17], [20]. In contrast, species richness of bees and wasps remained roughly constant. However, bees are known to be more species rich in warm and dry regions than in the wet tropics [22], [23]. The causes determining these richness patterns are still unclear and may involve a variety of current ecological and historical factors. Among the former, we hypothesized that food availability should be more closely related to species richness of our study groups than purely climatic factors.

In accordance with this hypothesis, food availability (number of flowers) showed a stronger relationship to species numbers of bees and wasp and hummingbirds than climatic factors (Table 4). The species numbers of both pollinator groups were correlated to the flower number of food plants, but only the number of bee and wasp species was also related to the species number of their food plants. For hummingbirds, this corresponds to previous conjectures based on local-scale studies that the abundance of flowers is more important in determining species numbers than the diversity of food plants [25], [65]. For bees, both the number of flowers and the number of plant species determine the species richness in Mediterranean habitats in Israel [30]. Further, food resources of the previous year, i.e., available to the bees as larvae, are stronger determinants of the species numbers of bees than the flowers availability in the sampling year [30], [66]. The concordance of our results with these previous studies is remarkable when we take into account that we also included wasp species into our study, which in contrast to bees mainly depend on insects and spiders as larval food. In this case, a direct link to the flowers only exists via the adults.

Butterflies, whose species richness was more closely related to climate than to flower numbers, take much of their energy in the adult stage from the sun [67] and feed exclusively on non-floral plant tissues as larvae. They are thus less dependent on flowers than bees or hummingbirds. It is therefore not surprising that we did not find a significant relationship between the species richness of butterflies and any flower-related factors. Only few studies [68], [69] have so far been able to demonstrate that nectar resources have a significant effect on the diversity or abundance of adult butterflies, either for single species or for entire communities. Due to their physiological conditions, butterflies are the only major pollinator group mainly affected by climatic factors such as minimum temperatures or the number of arid months, both in our study and in others focusing on single localities [70], [71] or along climatic gradients [17].

Comparing the species richness and abundance of pollinator groups between seasons, we found – contrary to our second hypothesis – no consistent seasonal shifts in species richness of bees and wasps, and very weak ones in hummingbirds. Butterflies, however, had significantly higher species richness as well as more unique species per site during the dry season. Butterflies often spend the dry season as adults and begin with reproduction at the beginning of the rainy season [72], [73]. Thus, the death of the adults of several species after reproduction and an increased mortality of larvae due to heavy rainfall [74] could be reasons for the impoverished assemblages found in our study during large parts of the rainy season. In addition, since the distribution of many tropical butterfly species is limited by frost [17], frost events in the nearby Andes during the dry season [75] led to downward movements of butterfly species, increasing the diversity of the local assemblages in that season. Similar avoidance movements are known for numerous tropical and extra-tropical butterfly species [76], [77]. Other studies found higher butterfly species numbers during the dry season [78], [79], higher species numbers during the rainy season [33] or no difference between seasons [32], [80]. Thus, Pozo et al. [71] concluded from the divergent results that butterfly richness is often determined by local factors such as microclimate.

When we compared the proportions of species numbers of the different pollinator groups and their food plants along the climatic gradient, in contrast to our third hypothesis we found no changes in the proportions of the pollinator and plant groups. Therefore, the potential benefit of the insect pollinator groups through their ability to survive periods with unfavourable food supply and weather conditions in larval stages or in their hives did not increase their relative species numbers at the climatically more seasonal sites. Hummingbirds, depending on a constant high availability of flowers [29], have apparently found a way to deal with the low amount of flowers during the dry season in the seasonal regions. We found markedly different assemblages of hummingbirds in each of the two southernmost localities Corbalán and Río Seco between the seasons. As known for honeyeaters in Australia [81], hummingbirds in the seasonal regions of Bolivia probably track the flowering season of plant species appropriate to their requirements by conducting nomadic movements. Cotton [24] already proposed that local migrations occur in some Amazonian hummingbird species. This would explain why the most abundant species in Corbalán during the rainy season, *Thalurania furcata*, was virtually absent during the dry season. *Thalurania* was replaced by *Chlorostilbon aureoventris*, a smaller hummingbird species that is able to efficiently utilize the tiny flowers of mass flowering *Tripodanthus acutifolius,* a primarily bee-pollinated Loranthaceae [29].

In conclusion, we found that the species numbers of the three groups of pollinators each showed different patterns along the climatic gradient and were correlated to different environmental factors. Combining our results with those from other studies, the factors correlating with the diversity of the different pollinator groups appear to be the same worldwide, regardless of the habitat type or the geographic latitude. Thus, on the local and regional scale, the number of bee and wasp species covaries with the availability and diversity of food resources in mediterranean or temperate openland habitats [30], [65] as well as in tropical and subtropical forests (our study). On a large scale, climatic aspects play a role in the diversity at least of bees [22], [23]. Hummingbird diversity is mainly correlated to the continuous availability of food regardless of the geographic latitude [25], [28]. Instead, butterfly diversity peaks in constantly humid, warm areas both worldwide [17] and on a regional scale (our study). Furthermore, not only the factors correlated to pollinator group diversity are the same worldwide, but in our study also the proportion in species numbers between pollinator groups remained constant along our gradient from subtropical, deciduous forests to tropical, evergreen rainforests. A similar tendency but with some variation was for some Caribbean islands [18] where pollinator diversity is reduced. Our results are especially surprising because the diversity of the different groups is related to different environmental factors.

Climate change will likely directly affect all pollinators by an increase in climatic seasonality and, perhaps more importantly, indirectly by changes in the distributions and phenologies of food plants [9]. In our study system, this is especially likely for hummingbirds, bees, and wasps, which we found to be strongly dependent on their food plants. In trophic networks were single plant species fulfil keystone roles at specific times, such changes may conceivably lead to massive disruptions of pollinator networks [82], when periods of insufficient food supply that may lead to decreases in pollinator diversity and abundance.

## Supporting Information

Table S1 Strength of relation (R-values) between the individual number of bees and wasp and hummingbirds total against biotic and abiotic environmental factors during rainy (R) and dry season (D) and total; ^ p<0.1, * p≤0.05.
(DOC)

Table S2 Strength of relation (R-values) for regression analysis between soil parameters and the number of pollinator group species and individuals of the different pollinator groups respectively number of food plant species and flowers during rainy (R) and dry season (D) and total; ^ p<0.1, * p≤0.05.
(DOC)

Table S3 Strength of relation (R-values) of linear regression analyses of the difference in species and individual numbers between seasons of the three pollinator groups and factors of climate and food seasonality; R = rainy season, D = dry season; ^ p<0.1, * p≤0.05.
(DOC)
