# Supplementary material for: The Influence of Climatic Seasonality on the Diversity of Different Tropical Pollinator Groups
Source: PLoS One. 2011 Nov 2;6(11):e27115. doi: 10.1371/journal.pone.0027115 (PMC3206942; doi:10.1371/journal.pone.0027115)
Supplement: Table S1 — Strength of relation (R-values) between the individual number of bees and wasp and hummingbirds total against biotic and abiotic environmental factors during rainy (R) and dry season (D) and total; ^ p<0.1, * p≤0.05. (DOC) [file pone.0027115.s001.doc]

Table S1: Strength of relation (R-values) between the individual number of bees and wasp and hummingbirds total against biotic and abiotic environmental factors during rainy (R) and dry season (D) and total; ^ p < 0.1, * p < 0.05.

|  | Bee and wasp individual No. | | | Hummingbird individual No. | | |
| --- | --- | --- | --- | --- | --- | --- |
|  | R | D | total | R | D | total |
| Elevation (m) | -0.53 | -0.17 | -0.36 | -0.04 | -0.48 | -0.20 |
| Annual precipitation (mm) | -0.22 | -0.48 | -0.48 | 0.10 | 0.16 | 0.37 |
| Mean temperature (°C) | 0.09 | -0.47 | 0.34 | 0.14 | 0.17 | 0.17 |
| Temperature amplitude (°C) | 0.12 | **0.76^** | 0.66 | -0.40 | -0.44 | -0.60 |
| No. of arid months | 0.02 | **0.74^** | 0.60 | -0.62 | -0.37 | -0.68 |
| Minimum temperature (°C) | -0.03 | -0.71 | -0.58 | 0.44 | 0.29 | 0.65 |
| Plant sp. R | 0.15 | -0.14 | -0.04 | 0.18 | 0.31 | 0.06 |
| Plant sp. D | **-0.82*** | 0.14 | -0.24 | 0.30 | 0.03 | 0.64 |
| Plant sp. total | -0.20 | 0.36 | 0.20 | 0.16 | 0.04 | 0.36 |
| Flower No. R | 0.68 | -0.09 | 0.23 | **0.87*** | 0.45 | **0.91*** |
| Flower No. D | 0.33 | **0.82*** | **0.80^** | 0.62 | 0.66 | 0.38 |
