# Supplementary material for: The Influence of Climatic Seasonality on the Diversity of Different Tropical Pollinator Groups
Source: PLoS One. 2011 Nov 2;6(11):e27115. doi: 10.1371/journal.pone.0027115 (PMC3206942; doi:10.1371/journal.pone.0027115)
Supplement: Table S2 — Strength of relation (R-values) for regression analysis between soil parameters and the number of pollinator group species and individuals of the different pollinator groups respectively number of food plant species and flowers during rainy (R) and dry season (D) and total; ^ p<0.1, * p≤0.05. (DOC) [file pone.0027115.s002.doc]

Table S2: Strength of relation (R-values) for regression analysis between soil parameters and the number of pollinator group species and individuals of the different pollinator groups respectively number of food plant species and flowers during rainy (R) and dry season (D) and total; ^ p < 0.1, * p < 0.05.

|  | Soil-pH | Cationic exchange capacity | Base saturation | C/N |
| --- | --- | --- | --- | --- |
| Bee and wasp sp. total | 0.02 | 0.37 | 0.07 | 0.35 |
| Bee and wasp sp. R | -0.13 | 0.18 | -0.11 | 0.39 |
| Bee and wasp sp. D | **0.81^** | 0.45 | **0.81*** | -0.10 |
| Butterfly sp. total | -0.28 | 0.31 | -0.32 | 0.60 |
| Butterfly sp. R | -0.55 | -0.22 | -0.65 | 0.36 |
| Butterfly sp. D | -0.19 | 0.35 | -0.23 | 0.51 |
| Hummingbird sp. total | -0.33 | 0.20 | -0.28 | 0.36 |
| Hummingbird sp. R | -0.36 | 0.25 | -0.30 | 0.38 |
| Hummingbird sp. D | -0.49 | 0.07 | -0.40 | 0.33 |
| Bee and wasp ind. total | 0.15 | 0.22 | 0.36 | 0.41 |
| Bee and wasp ind. R | -0.17 | 0.10 | 0.05 | -0.21 |
| Bee and wasp ind. D | 0.28 | 0.23 | 0.43 | -0.40 |
| Hummingbird ind. total | -0.04 | 0.46 | -0.02 | 0.50 |
| Hummingbird ind. R | 0.03 | 0.59 | 0.08 | 0.30 |
| Hummingbird ind. D | -0.10 | 0.28 | -0.13 | 0.65 |
| Bee and wasp plant sp. total | -0.12 | -0.11 | 0.05 | -0.82* |
| Bee and wasp plant sp. R | -0.68 | -0.08 | -0.53 | -0.25 |
| Bee and wasp plant sp. D | -0.12 | -0.34 | -0.21 | -0.15 |
| Butterfly plant sp. total | -0.46 | -0.54 | -0.32 | -0.57 |
| Butterfly plant sp. R | **0.82*** | -0.54 | -0.65 | -0.39 |
| Butterfly plant sp. D | -0.34 | -0.60 | -0.36 | -0.15 |
| Hummingbird plant sp. total | **-0.82*** | -0.35 | -0.65 | -0.14 |
| Hummingbird plant sp. R | **-0.81^** | 0.42 | -0.65 | -0.23 |
| Hummingbird plant sp. D | **-0.88*** | -0.39 | -**0.77^** | 0.15 |
| Bee and wasp flower R | -0.12 | 0.49 | -0.03 | 0.26 |
| Bee and wasp flower D | 0.53 | 0.58 | 0.60 | -0.01 |
| Butterfly flower R | -0.61 | 0.02 | -0.42 | -0.07 |
| Butterfly flower D | 0.48 | 0.50 | 0.54 | 0.01 |
| Hummingbird flower R | -0.29 | 0.24 | -0.11 | 0.01 |
| Hummingbird flower D | 0.25 | 0.61 | 0.38 | 0.03 |
