# Supplementary material for: The Influence of Climatic Seasonality on the Diversity of Different Tropical Pollinator Groups
Source: PLoS One. 2011 Nov 2;6(11):e27115. doi: 10.1371/journal.pone.0027115 (PMC3206942; doi:10.1371/journal.pone.0027115)
Supplement: Table S3 — Strength of relation (R-values) of linear regression analyses of the difference in species and individual numbers between seasons of the three pollinator groups and factors of climate and food seasonality; R = rainy season, D = dry season; ^ p<0.1, * p≤0.05. (DOC) [file pone.0027115.s003.doc]

Table S3: Strength of relation (R-values) of linear regression analyses of the difference in species and individual numbers between seasons of the three pollinator groups and factors of climate and food seasonality; R = rainy season, D = dry season; ^ p < 0.1, * p < 0.05.

|  | No. of arid months | Temperature amplitude | Food plant spec. No. R-D | Flower no.  R-D |
| --- | --- | --- | --- | --- |
| Bee and wasp spec. no. R-D | 0.47 | 0.47 | 0.31 | -0.19 |
| Bee and wasp ind. no. R-D | -0.72 | -0.68 | 0.47 | 0.79^ |
| Butterfly spec. no. R-D | 0.73 | 0.55 | -0.07 | 0.37 |
| Hummingbird spec. no. R-D | -0.81* | -0.58 | 0.18 | 0.30 |
| Hummingbird ind. no. R-D | -0.22 | 0.21 | -0.05 | 0.28 |
